# Household Air Pollution and Dementia Risk in Low‐ and Middle‐Income Countries ‐A Systematic Review of literature

**DOI:** 10.1002/alz.085892

**Published:** 2025-01-09

**Authors:** Suvarna Jyothi Kantipudi

**Affiliations:** ^1^ UNIVERSITY OF CALIFORNIA, BERKELEY, California, CA, USA; Department of Psychiatry, SRIHER, Chennai India

## Abstract

**Background:**

Exposure to the household air pollution (HAPIN) is a leading health risk in populations in LMIC s and accounts for an estimated 2.3 million premature deaths annually and 91.5 million disability‐adjusted life years (Bennitt et al,2021). While the deleterious effects of household air pollution on respiratory and cardiovascular health are well‐documented, the impact of HAP on cognitive outcomes, specifically dementia risk, remains an area of evolving research. Previous systematic reviews have highlighted associations between air pollution, especially particulate matter (PM) and neurodegenerative conditions in high‐income countries, and limits the generalizability of findings to LMICs, where unique patterns of air pollution exposure and sociodemographic factors may play crucial roles. In this context, the current study aims to contribute to the existing body of knowledge by systematically reviewing and synthesizing evidence on household air pollution exposures and their potential association with dementia risk in LMICs.

**Method:**

A systematic search of databases for studies linking household air pollution (HAP) to dementia in low‐ and middle‐income countries will be conducted. Inclusion criteria are human studies published in peer‐reviewed journals in PubMed and Embase databases. Extracted data will include study characteristics, exposure assessments, and cognitive outcomes.

**Result:**

The systematic review is currently ongoing, and initial searches identified a substantial number of relevant studies exploring the association between household air pollution (HAP) and dementia in low‐ and middle‐income countries. Data extraction revealed diverse study designs, geographical locations, and cognitive outcome measures. Subsequent narrative synthesis will provide a comprehensive overview of the existing evidence, elucidating patterns and potential sources of heterogeneity. Subgroup analyses will be done to understand regional differences and socio‐demographic factors, contributing to a nuanced understanding of HAP’s impact on dementia risk.

**Conclusion:**

While the systematic review is ongoing, its anticipated outcomes hold promise for advancing our understanding of the complex relationship between household air pollution and dementia in low‐ and middle‐income countries.

